# Image diagnosis models for the oral assessment of older people using convolutional neural networks: A retrospective observational study

**DOI:** 10.1111/jocn.16182

**Published:** 2021-12-21

**Authors:** Misato Muramatsu, Masumi Muramatsu, Naoto Takahashi, Atsuko Hagiwara, Jyun Hagiwara, Yuichiro Takamatsu, Ryo Morooka, Morio Ochi, Toshiko Kaitani

**Affiliations:** ^1^ Graduate School of Nursing Sapporo City University Sapporo Japan; ^2^ School of Nursing Sapporo City University Sapporo Japan; ^3^ AI Laboratory Sapporo City University Sapporo Japan; ^4^ Hagiwara Dental Clinic Ashibetsu Japan; ^5^ Takamatsu Dental Office Ishikari Japan; ^6^ Akaiwa Ryo Dental Clinic Otaru Japan; ^7^ Division of Fixed Prosthodontics and Oral Implantology Department of Oral Rehabilitation School of Dentistry Health Sciences University of Hokkaido Ishikari‐Tobetsu Japan

**Keywords:** artificial intelligence, convolutional neural network, deep learning, dental hygiene, image diagnosis, nursing, older, oral assessment, oral assessment guide, oral care

## Abstract

**Aims:**

The purpose of this study was to construct a model for oral assessment using deep learning image recognition technology and to verify its accuracy.

**Background:**

The effects of oral care on older people are significant, and the Oral Assessment Guide has been used internationally as an effective oral assessment tool in clinical practice. However, additional training, education, development of user manuals and continuous support from a dental hygienist are needed to improve the inter‐rater reliability of the Oral Assessment Guide.

**Design:**

A retrospective observational study.

**Methods:**

A total of 3,201 oral images of 114 older people aged >65 years were collected from five dental‐related facilities. These images were divided into six categories (lips, tongue, saliva, mucosa, gingiva, and teeth or dentures) that were evaluated by images, out of the total eight items that comprise components of the Oral Assessment Guide. Each item was classified into a rating of 1, 2 or 3. A convolutional neural network, which is a deep learning method used for image recognition, was used to construct the image recognition model. The study methods comply with the STROBE checklist.

**Results:**

We constructed models with a classification accuracy of 98.8% for lips, 94.3% for tongue, 92.8% for saliva, 78.6% for mucous membranes, 93.0% for gingiva and 93.6% for teeth or dentures.

**Conclusions:**

Highly accurate diagnostic imaging models using convolutional neural networks were constructed for six items of the Oral Assessment Guide and validated. In particular, for the five items of lips, tongue, saliva, gingiva, and teeth or dentures, models with a high accuracy of over 90% were obtained.

**Relevance to Clinical Practice:**

The model built in this study has the potential to contribute to obtain reproducibility and reliability of the ratings, to shorten the time for assessment, to collaborate with dental professionals and to be used as an educational tool.

**What does this paper contribute to the wider global clinical community?**

- We demonstrated that a convolutional neural network can be used to construct a diagnostic imaging model for the oral assessment of older people, and we obtained a highly accurate model.
- The model built in this study has the potential to contribute to clinical nursing practice, such as obtaining reproducibility and reliability of the ratings, shortening the time for oral assessment, collaborating with dental professionals and being used as an educational tool.

## 1 INTRODUCTION

Hospitalisation due to pneumonia in older people causes muscle impairment and increases the risk of cognitive decline (Martín‐Salvador et al., 2015; Shah et al., 2013). Oral care for older people is reported to reduce the risk of contracting fever and pneumonia (Yoneyama et al., 1996, 1999, 2002). Furthermore, oral care using devices can improve the quality of life (QOL) of older people in long‐term care senior residential facilities (Riggs et al., 2020). Therefore, the oral healthcare regimens of older people play a very important role in maintaining their health.

## 2 BACKGROUND

The Oral Assessment Guide (OAG) has been considered a reliable and effective tool to evaluate oral status and function, and it is used internationally, especially in clinical practice (Eilers et al., 1988; Holmes & Mountain, 1993). However, it is suggested that additional training, education, development of user manuals and continuous support from a dental hygienist are needed to improve the inter‐rater reliability of OAG (Andersson et al., 2002; Aoki et al., 2019).

The development of artificial intelligence (AI) technology has led to its application in a variety of fields. Deep learning, an AI function that imitates the workings of the human brain in processing data, allows computational models composed of multiple processing layers to learn the representation of data with multiple levels of abstraction (LeCun et al., 2015). In particular, convolutional neural networks (CNNs), regarded as a subset of deep learning, have demonstrated advanced image recognition capabilities (Krizhevsky et al., 2017). Recent research has also been done on their application in a variety of fields, including clinical settings. In the medical field of dermatology, CNN was able to classify skin cancer with a level of competence comparable to dermatologists (Esteva et al., 2017). In addition, a study conducted with a large number of dermatologists showed that the model constructed using CNN could identify melanoma with higher accuracy than dermatologists could (Haenssle et al., 2018). In the field of dentistry, the CNN algorithm has been effective in detecting and diagnosing dental caries from periapical radiographs (Lee et al., 2018). While it can be concluded that diagnostic imaging models using CNNs are useful for clinical practice, no studies on the construction of models using CNNs have been reported in the field of nursing and oral health.

The purpose of this study was to construct a model for oral assessment using CNNs, a method of deep learning image recognition technology, and to verify its accuracy. By achieving this objective, we can resolve the problems behind applying OAG to practical fields, which will ultimately contribute to the prevention of hospitalisation caused by pneumonia and the improvement of QOL for older people.

## 3 METHODS

### 3.1 Design

The design in this study is a retrospective observational study. The study methods comply with the strengthening the reporting of observational studies in epidemiology statement (Data S1).

### 3.2 Participants

We collected 3,201 oral images from 114 consenting older people, aged 65 years or older, from five dental‐related facilities. We approached dental facilities, where the older people gathered, in the neighbouring areas and, based on the snowball sampling method, obtained a sample comprising the facilities that were willing to cooperate. We identified five facilities that routinely took images as part of their practice and could provide an adequate number of oral images for analysis. Older people were randomly selected.

### 3.3 Data collection

The retrospective collection period was from November 2012 to April 2021. The device used to take the pictures did not matter for this study.

### 3.4 Ethical considerations

This study was based on the Ethical Guidelines for Medical and Health Research Involving Human Subjects issued by the Japanese Ministry of Health, Labour and Welfare, and approved by the Sapporo City University Graduate School of Nursing Ethical Review Committee (No. 10, 2020). We obtained written informed consent from the participants. When we used images collected before this study had been started, participants were informed of their options for opting out of the study through the Internet and notices in the facilities.

### 3.5 Data analysis

#### 3.5.1 OAG

The assessment tool used as a base in this study was the OAG, which has been validated in clinical practice (Eilers et al., 1988; Holmes & Mountain, 1993).

**FIGURE 1:**
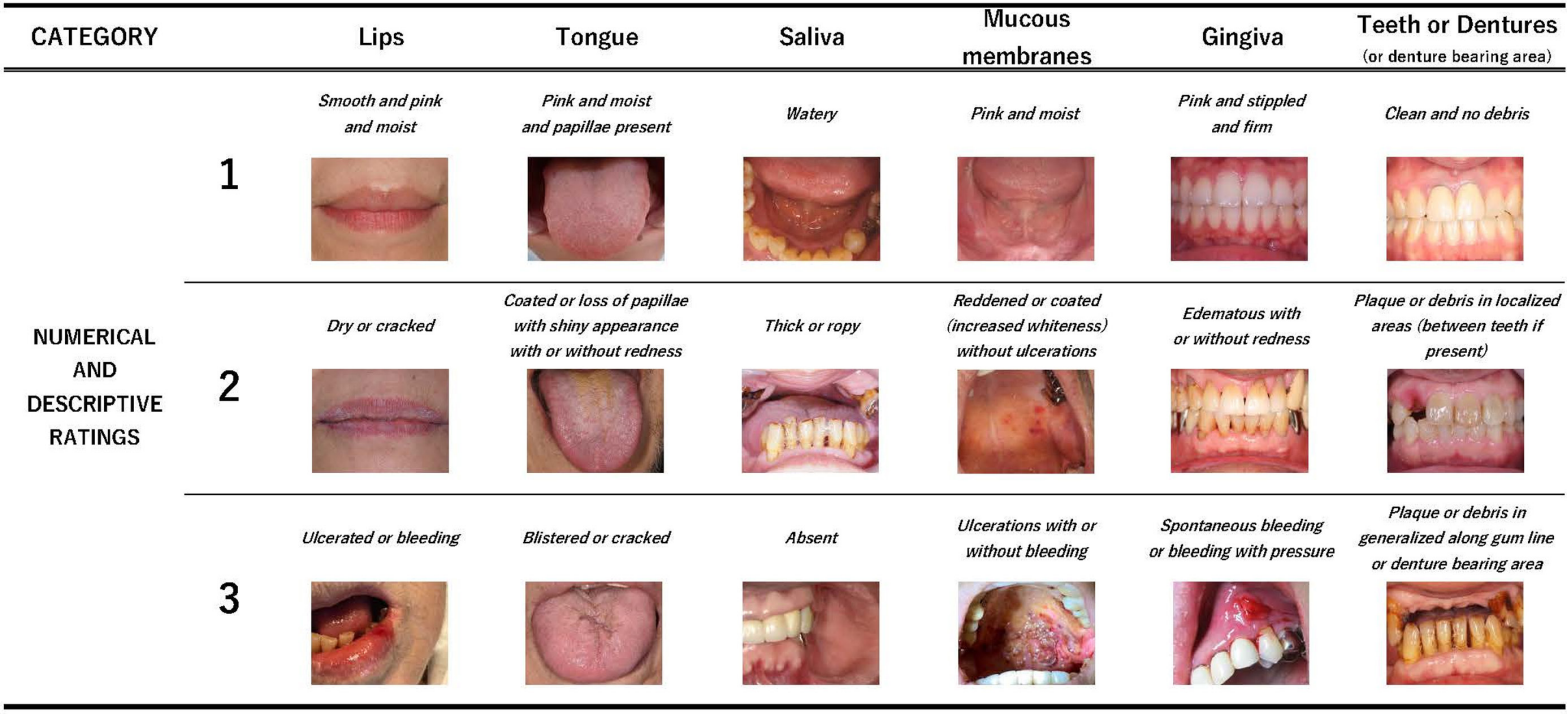
Examples of the OAG elements used in this study. The images were classified into ratings of 1 to 3 for each category. The table shows the representative examples used in this study [Colour figure can be viewed at wileyonlinelibrary.com]

The OAG consists of eight items, of which we focused on six items (lips, tongue, saliva, mucous membranes, gingiva, and teeth or dentures) that could be evaluated visually. These results are summarised in Figure 1, along with the example images used in this study. We evaluated the condition of the lips as smooth and pink and moist for rating 1, dry or cracked for rating 2 and ulcerated or bleeding for rating 3; the condition of the tongue as pink and moist and papillae present for rating 1, coated or loss of papillae with shiny appearance with or without redness for rating 2 and blistered or cracked for rating 3; the condition of the saliva as watery for rating 1, thick or ropy for rating 2 and absent for rating 3; the condition of the mucous membranes as pink and moist for rating 1, reddened or coated (increased whiteness) without ulcerations for rating 2 and ulcerations with or without bleeding for rating 3; the condition of the gingiva as pink and stippled and firm for rating 1, oedematous with or without redness for rating 2 and spontaneous bleeding or bleeding with pressure for rating 3; the condition of the teeth or dentures (or denture bearing area) as clean and no debris for rating 1, plaque or debris in localised areas (between teeth if present) for rating 2 and plaque or debris in generalised along gum line or denture bearing area for rating 3.

In our study, categorisation was defined as the division of six items in the OAG, and classification referred to the numerical ratings of 1 to 3 based on the descriptive rating of the images in each OAG category.

#### 3.5.2 CNN

To analyse the images shown in Figure 1, we adopted a CNN, which is a supervised deep learning algorithm, which is widely used to analyse visual images.

The CNN model was created by combining one or more of the following: a convolution layer, a pooling layer and a fully connected layer that extracted features from the input, minimised the size for computational performance and classified the image accordingly.

Python (version 3.7) was used to create the CNN program. TensorFlow (version 2.0) and Keras (version 2.3) were used as libraries for deep learning.

### 3.6 Procedures

#### 3.6.1 Sample size for categorisation and classification

**FIGURE 2:**
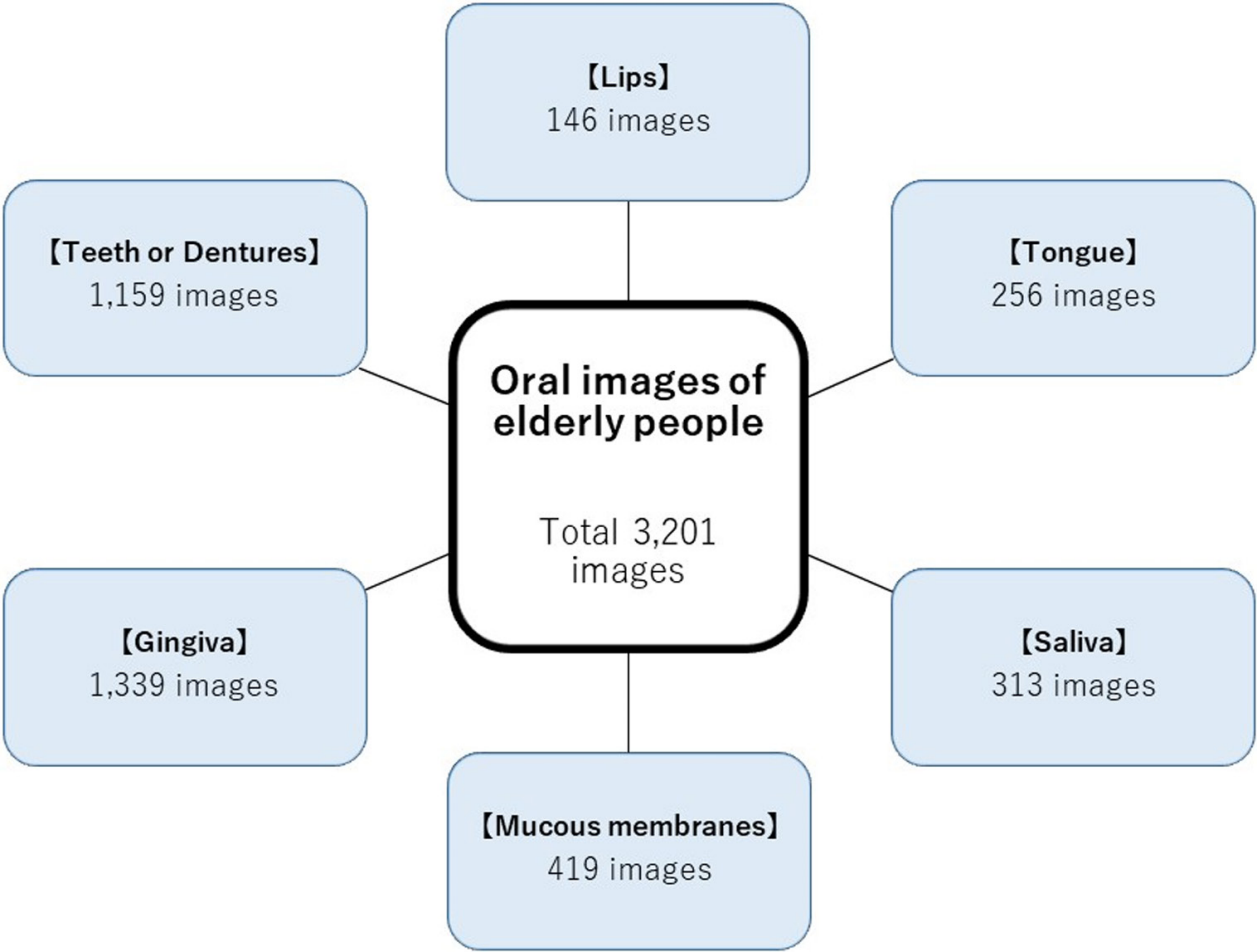
Images categorisation. We categorised the oral images of older people into six items that clearly showed the object, and that could be identified. In some cases, however, each image contains more than one object, such that the images after categorisation are duplicates of the original images [Colour figure can be viewed at wileyonlinelibrary.com]

The 3,201 images were categorised into six items each, provided the objects were clearly visible and distinguishable. They were categorised into 146 lips, 256 tongues, 313 saliva samples, 419 mucous membranes, 1,339 gingivae and 1,159 teeth or dentures. In some cases, however, each image contained more than one object, such that the images after categorisation were duplicates of the original images (Figure 2). Thereafter, each category was classified into a rating of 1, 2 or 3.

#### 3.6.2 Image adjustment and analysis

The object images were trimmed if there were parts of the images that were not relevant to the assessment. In addition, data augmentation was conducted because training a small amount of data leads to overfitting and poor generalisation performance of the model.

Eighty per cent of the images classified into each rating were used for training and the rest for testing. The model was constructed, and its accuracy was verified. In building the model, the number of epochs and image sizes were tuned to minimise validation loss. The model with the smallest validation loss was used to classify the images for validation and test the accuracy.

In addition, we compared the original diagnosis by the expert and the diagnosis determined by AI using the constructed CNN model in a confusion matrix.

### 3.7 Validity, reliability and rigour

The categorisation and classification were performed by three nurses with more than eight years of clinical experience, including a certified instructor of the Japanese Academic Society for Oral Care, to ensure reliability and validity.

## 4 RESULTS

### 4.1 Classification and adjustment of the number of images for each category

#### 4.1.1 Lips

**FIGURE 3:**
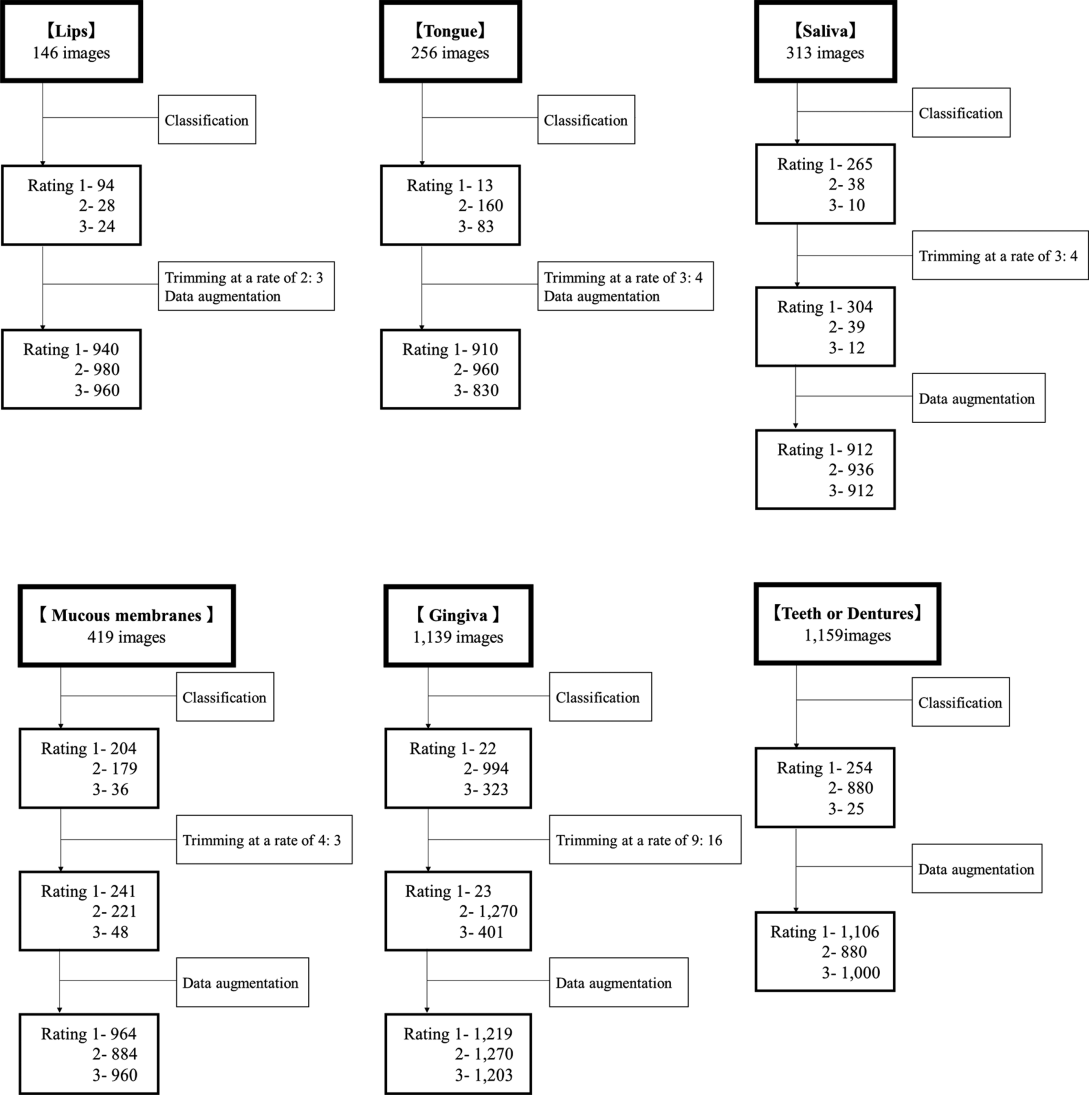
Classification and adjustment. The number of images classified in each category and the procedure used to adjust the number of images in the analysis professionally

The 146 images were classified into 94 images of rating 1, 28 images of rating 2 and 24 images of rating 3 (Figure 3). Images with parts not related to the evaluation were trimmed at a ratio of 2:3, to identify the object of analysis, and were then finally adjusted by data augmentation to 940 with a rating of 1, 980 with a rating of 2 and 960 with a rating of 3.

#### 4.1.2 Tongue

The 256 images were classified into 13 images of rating 1, 160 images of rating 2 and 83 images of rating 3. Images with parts not related to the evaluation were trimmed at a ratio of 3:4, to identify the object of analysis, and were then finally adjusted by data augmentation to 910 with a rating of 1, 960 with a rating of 2 and 830 with a rating of 3.

#### 4.1.3 Saliva

The 313 images were classified into 265 images of rating 1, 38 images of rating 2 and 10 images of rating 3. Images with parts not related to the evaluation were trimmed at a ratio of 3:4, to identify the object of analysis. In some cases, it was possible to obtain more than one feature of the analysis target from a single image, so the number of images was increased to 304 with a rating of 1, 39 with a rating of 2 and 12 with a rating of 3. Then, the data were finally adjusted by data augmentation, to 912 with a rating of 1, 936 with a rating of 2 and 912 with a rating of 3.

#### 4.1.4 Mucous membranes

The 419 images were classified into 204 images of rating 1, 179 images of rating 2 and 36 images of rating 3. Images with parts not related to the evaluation were trimmed at a ratio of 4:3, to identify the object of analysis. In some cases, it was possible to obtain more than one feature of the analysis target from a single image, so the number of images was increased to 241 with a rating of 1, 221 with a rating of 2 and 48 with a rating of 3. The data were finally adjusted by data augmentation to 964 with a rating of 1, 884 with a rating of 2 and 960 with a rating of 3.

#### 4.1.5 Gingiva

The 1,339 images were classified into 22 images of rating 1, 994 images of rating 2 and 323 images of rating 3. Images with parts not related to the evaluation were trimmed at a ratio of 9:16, to identify the object of analysis. In some cases, it was possible to obtain more than one feature of the analysis target from a single image, so the number of images was increased to 23 with a rating of 1, 1,270 with a rating of 2 and 401 with a rating of 3. Then, ratings 1 and 3 were adjusted by data augmentation to match the number of images in rating 2, and the data were finally adjusted to 1,219 with a rating of 1, 1,270 with a rating of 2 and 1,203 with a rating of 3.

#### 4.1.6 Teeth or dentures

The 1,159 images were classified into 254 images of rating 1, 880 images of rating 2 and 25 images of rating 3. Images of this category were not trimmed because there were a few cases where parts of the images were not relevant to the analysis, and the subject of the analysis was clearly visible. Ratings 1 and 3 were adjusted by data augmentation to match the number of images in rating 2, and the data were finally adjusted to 1,016 with a rating of 1, 880 with a rating of 2 and 1,000 with a rating of 3.

### 4.2 Accuracy of the model

#### 4.2.1 Lips

**FIGURE 4:**
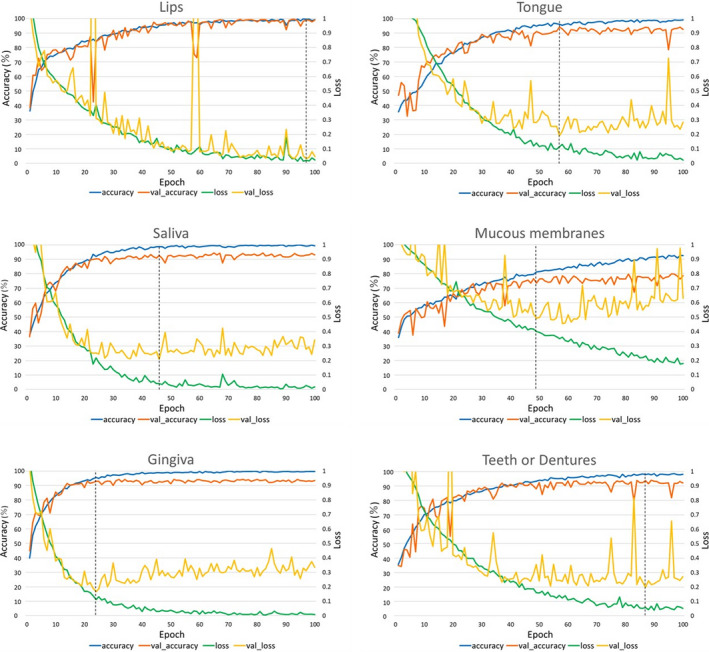
Comparison of the accuracy and loss. Accuracy and loss of the training and validation data for each category, and the relationship with the number of training times. The “accuracy” is the percentage of correct answers in the training data, and the “val_accuracy” is the percentage of correct answers in the validation data. Furthermore, “loss” represents the loss in the training data, and “val_loss” represents the loss in the validation data. The val_accuracy with the smallest val_loss in each category was considered as the accuracy of the model [Colour figure can be viewed at wileyonlinelibrary.com]

The image size was 20 × 30 pixels, and the validation loss was minimised to 0.033 at 97 training epochs (Figure 4). After accuracy verification using the validation image, the classification accuracy was 98.8%.

#### 4.2.2 Tongue

The image size was 30 × 40 pixels, and the validation loss was minimised to 0.190 at 57 training epochs. After accuracy verification using the validation image, the classification accuracy was 94.3%.

#### 4.2.3 Saliva

The image size was 30 × 40 pixels, and the validation loss was minimised to 0.209 at 46 training epochs. After accuracy verification using the validation image, the classification accuracy was 92.8%.

#### 4.2.4 Mucous membranes

The image size was 20 × 15 pixels, and the validation loss was minimised to 0.455 at 58 training epochs. After accuracy verification using the validation image, the classification accuracy was 78.6%.

#### 4.2.5 Gingiva

The image size was 27 × 48 pixels, and the validation loss was minimised to 0.167 at 24 training epochs. After accuracy verification using the validation image, the classification accuracy was 93.0%.

#### 4.2.6 Teeth or Dentures

The image size was 15 × 20 pixels, and the validation loss was minimised to 0.199 at 87 training epochs. After accuracy verification using the validation image, the classification accuracy was 93.6%.

### 4.3 Correct answer percentages

**FIGURE 5:**
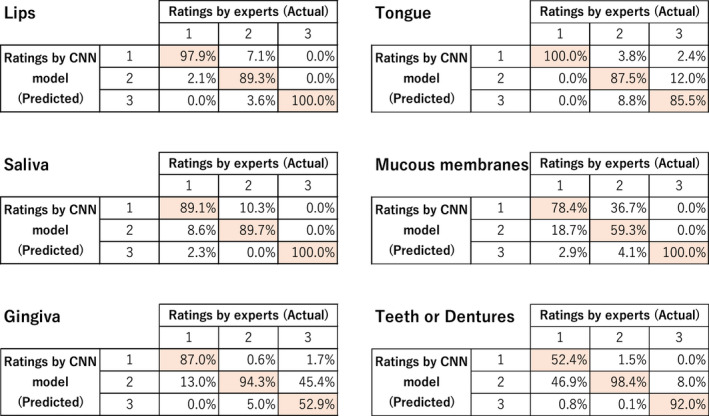
Correct answer percentages. For each category, the evaluation by the expert is expressed as the actual, the assessment judged by the AI using the CNN model as the predicted, and the rate of correct answers as the percentages [Colour figure can be viewed at wileyonlinelibrary.com]

We present the results obtained from comparing the original diagnosis ratings by experts with the ratings determined in AI using the constructed CNN model by a confusion matrix (Figure 5). The original images were diagnosed, instead of the expanded image, because the model was built with expanded data, and the number of each score was varied; therefore, the results were expressed in percentages instead of numbers.

#### 4.3.1 Lips

The correct answer percentages of the CNN model for experts were 97.9% for rating 1, 89.3% for rating 2 and 100.0% for rating 3.

#### 4.3.2 Tongue

The correct answer percentages of the CNN model for experts were 100.0% for rating 1, 87.5% for rating 2 and 85.5% for rating 3.

#### 4.3.3 Saliva

The correct answer percentages of the CNN model for experts were 89.1% for rating 1, 89.7% for rating 2 and 100.0% for rating 3.

#### 4.3.4 Mucous membranes

The correct answer percentages of the CNN model for experts were 78.4% for rating 1, 59.3% for rating 2 and 100.0% for rating 3.

#### 4.3.5 Gingiva

The correct answer percentages of the CNN model for experts were 87.0% for rating 1, 94.3% for rating 2 and 52.9% for rating 3.

#### 4.3.6 Teeth or dentures

The correct answer percentages of the CNN model for experts were 52.4% for rating 1, 98.4% for rating 2 and 92.0% for rating 3.

## 5 DISCUSSION

### 5.1 Summary

In this study, we, for the first time, constructed and verified the accuracy of a model for oral assessment of six items that can be evaluated visually in OAG. Due to the CNN analysis by adjusting the image size and epochs, we were able to construct an image diagnosis model with high accuracy. In particular, we achieved an accuracy of over 90% for five categories other than mucous membranes: lips, tongue, saliva, gingiva, and teeth or dentures. Furthermore, the correct answer percentage of the CNN model for experts was approximately 90% to 100% for most of the categories.

### 5.2 Accuracy and correct answer percentages

The accuracy of the CNN model was relatively low only for mucous membranes; however, it still achieved approximately 80% accuracy. In the categories other than mucous membranes, it exceeded 90% accuracy. Therefore, we expect to be able to use the results for clinical diagnosis.

Regarding the percentage of correct answers, the ratings of 3 for gingiva and 1 for teeth and dentures were approximately 50%; however, the percentage of two‐step errors (diagnosing a rating of 1 as 3 or the opposite) was within the 0% to 2.9% range for all items, indicating that the AI did not make any significant errors in assessment. In addition, although the percentage of correct answers for 3 for gingiva and 1 for teeth and dentures was approximately 50%, we assume that the AI will be able to diagnose effectively in practice because the AI was able to distinguish between a normal score of 1 for gingiva and other conditions, and the probability of missing a bad condition for teeth and dentures was low.

### 5.3 Proposal for practical use in the nursing field

In the medical field, a paradigm for diagnosing anaemia non‐invasively, using only patient‐sourced photographs, has been reported as an effective and feasible smartphone application (Mannino et al., 2018). In recent years, studies have also summarised the effective use of mobile technology for nurses, patients and undergraduate nursing students in the nursing field (Silva et al., 2018). The development of easy‐to‐use mobile tools, such as applications for oral assessment using this model, is expected to contribute to wider nursing practice.

For example, a significant difference has been observed between professionals’ assessments and self‐assessments in older people who use short‐term care units, and the latter are at a higher risk of oral problems (Koistinen et al., 2020). To solve such cases, older people can personally use the mobile tool with CNN. Undoubtedly, staff members other than nurses, as well as family members, can also use it. Furthermore, it is hoped that its application expands to nursing homes and hospitals.

### 5.4 Limitations

The present study had a few limitations. First, the images collected in this study showed variations in the number of rating classifications for each category. In addition, this study focused on collecting images from higher‐risk older people, so age was not a universal factor in this case. In medical research using CNNs, the number of images used for deep learning varies in the range of ten to over 100,000 reports (Esteva et al., 2017; Hwang et al., 2019). In the next few years, the task is to increase the number of samples with fewer ratings by broadening the target population, to build a more comprehensive model, and improve the accuracy.

Second, only the accuracy of the mucous membrane classification model was unable to exceed 80%. The reasons for this may include not only the variation in the number of ratings, but also the possibility that the features of the photographs were not clearly identified. In particular, it was difficult to separate the different colours of mucous membranes due to the light conditions and to separate the capillaries and redness. Therefore, in future, we would like to improve the accuracy, not only by increasing the number of samples to minimise the variation, but also by collecting good‐quality images that are more likely to reveal features.

Third, the quality of the images may have been affected by the procedures (e.g. rinsing or blowing air) performed before the photographs were taken, especially for the categories of tongue, saliva and mucous membranes, which included the degree of moisture as a determining factor. When collecting images in future, it is necessary to take photographs before the procedure to maintain consistency with the actual oral condition.

Finally, this study only constructed models, verified their accuracy and did not demonstrate their practicality in clinical practice. In particular, we focused on six items that could be visually determined among all eight items of the OAG. Although the other items, voice and swallow, are considered relatively easy to obtain inter‐rater reliability on, it is necessary to conduct a practical evaluation including these two items. In medical research, AI diagnosis and specialist diagnosis are compared (Esteva et al., 2017; Haenssle et al., 2018). Therefore, it is necessary to confirm the effectiveness of this model by comparing the model constructed in this study with the diagnostic performance of clinical nurses or experts.

## 6 CONCLUSION

For the first time, we demonstrated that high accuracy models for oral assessments could be built using images by analysing CNNs. The results provide important implications for the nursing field in terms of using artificial intelligence to accurately assess oral issues in older people.

## 7 RELEVANCE TO CLINICAL PRACTICE

As previously mentioned, it has been established that training, education, user manuals and continuous support from a dental hygienist are needed to improve the inter‐rater reliability of OAG (Andersson et al., 2002; Aoki et al., 2019). In addition, oral care has been reported to play a peripheral role in nursing care, while it actually occupies an integral position in nursing homes for older people (Lindqvist et al., 2013).

The model we constructed in this study would be able to solve these guide‐related problems based on AI judgments. This is because the model can be used quickly and universally, not requiring any special training or education. Through our study, we were able to distinguish the features sufficiently, even with images taken using a smartphone. Therefore, the model has the potential to shorten the time of oral assessment and ensure the reproducibility and reliability of the ratings. If a uniform assessment can be performed accurately in a short time, the model can be used as a tool to aid dental professionals and as an educational tool for staff.

When the application reflecting the CNN model that has been built in this study is developed in future, we will need to ensure that it is simple to handle so that nurses and other healthcare staff can implement it smoothly. To be able to use the application as intended, it will be important for the nurses and healthcare staff to be knowledgeable about the possibility of oral problems in high‐risk patients, such as older people, patients undergoing chemotherapy or radiation therapy, and postoperative patients. Next, they will need to take a picture of the object so that it is large enough to be screened without being out of focus. Moreover, the picture should be taken in a bright place so that the colours can be easily identified. Lastly, it should become common practice to share the resulting information with the staff. By repeating this training cycle, the AI model can become a supportive and educational tool for healthcare professionals.

However, there are a few limitations. First, even if we improve the accuracy of the AI in future, it will not be 100%, and diagnoses cannot be completely dependent on machines. It will be necessary for nurses to keep educating themselves on oral assessment to overcome this problem. Second, the oral assessment in this study was performed using a machine that has never been used in practice before and which may take time to be adapted. Therefore, we would like to simplify the introduction and use of the application through a development process that includes user evaluation.

## CONFLICT OF INTEREST

The authors declare that they have no conflict of interest.

## AUTHOR CONTRIBUTION

MM, MM, NT, MO and TK were involved in the study design. MM, MM, AH, JH, JT, RM and MO were involved in the data collection. MM, MM and NT were involved in the data analysis and data interpretation. All authors critically revised the report, commented on drafts of the manuscript and approved the final report.

## Supporting information

Supplementary Material

## Data Availability

The data that support the findings of this study are available from the corresponding author upon reasonable request.
